# First documented cure of a suggestive exogenous reinfection in polymyositis with same but multidrug resistant *M. tuberculosis*

**DOI:** 10.1186/1471-2334-4-63

**Published:** 2004-12-23

**Authors:** Chiranjoy Mukhopadhyay, Ankita Garg, Archana Ayyagari

**Affiliations:** 1 Department of Microbiology, Sanjay Gandhi Postgraduate Institute of Medical Sciences, Raebarely Road, Lucknow, Uttar Pradesh, Pin: 226014, India; 2 Current address: Department of Microbiology, Manipal College of Medical Sciences, P.O. Box 155, Deep Heights 16, Pokhara, Nepal; 3 Corresponding address: Department of Microbiology, Manipal College of Medical Sciences, P.O. Box 155, Deep Heights 16, Pokhara, Nepal; 4 Department of Microbiology, Sanjay Gandhi Postgraduate Institute of Medical Sciences, Raebarely Road, Lucknow, Uttar Pradesh, Pin: 226014, India; 5 Department of Microbiology, Sanjay Gandhi Postgraduate Institute of Medical Sciences, Raebarely Road, Lucknow, Uttar Pradesh, Pin: 226014, India

## Abstract

**Background:**

MDR *Mycobacterium tuberculosis *is the major cause of treatment failure in tuberculosis patients, especially in immunosuppressed. We described a young polymyositis patient on immunosuppressive therapy who was started with antituberculosis therapy as a susceptible strain of *M. tuberculosis *was isolated from a single cutaneous abscess in his neck and from regional lymph nodes.

**Case presentation:**

He had non-reactive miliary tuberculosis and multiple cutaneous abscesses 6 months later with the same strain, which was resistant this time to 9 antituberculosis drugs. We described clinical presentation, radiological and laboratory work-up, treatment and follow-up as the patient was cured after 1.5 years with 6 antituberculosis drugs.

**Conclusion:**

To our knowledge, this is the first reported case where an immunosuppressed patient with suggestive exogenous reinfection within 6 months with the same but MDR strain of *M. tuberculosis *was cured. Intense management and regular follow up were important since the patient was a potent source of MDR *M. tuberculosis *infection and there was limited choice for therapy.

## Background

World Health Organization published the Global Tuberculosis Control Report (2003) on 'World Tuberculosis Day' where India is ranked number one in the world for high incidence of smear positive cases of pulmonary (about 0.9 million) and extrapulmonary TB (about 0.2 million) every year [1]. Undoubtedly, TB itself has reemerged in India as a serious problem since 1985 with the advent of HIV/AIDS [2]. However, cutaneous TB is still rare in countries like India (0.10%), Hong Kong (0.07%) and Madrid (0.14%) [2] and it manifests either as a true bacterial invasion or as a tuberculid (hypersensitivity reaction) with primary focus elsewhere. Evidences of bacterial invasion are found in lupus vulgaris, the most common manifestation (55%) in patients with cutaneous TB (1975–95) in northern India [2], primary chancre, tuberculous verrucosa cutis, scrofuloderma, tuberculous cutis orificialis and tuberculous cutis miliaris disseminates [3] whereas erythema induratum (Bazin disease) and lichen scrofulosorum are tuberculid lesions [2]. Moreover, atypical mycobacteria such as *M. kansasii*, *M. scrofulaceum*, rather than *M. tuberculosis *are the most common etiological agents for cutaneous TB in HIV/AIDS and other immunocompromised patients.

WHO defines acquired drug resistance as the isolation of drug-resistant *M. tuberculosis *from a patient who has been treated for TB for 1 month or longer [4] and primary drug resistance as the isolation of drug-resistant strain from a patient without a history of previous treatment. The selection of *M. tuberculosis *with mutations conferring resistance to antitubercular drugs may result from poor management including the prescription of incorrect regimens and non-compliance with treatment.

We report a rare case of reinfection with same but MDR *M. tuberculosis *in a polymyositis patient on immunosuppressive therapy resulting in multiple cutaneous abscesses and miliary TB.

## Case Presentation

A 23-year-old polymyositis patient with suggestive clinical symptoms, muscle biopsy, enzyme assay, needle EMG and NCV test was on immunosuppressive drugs such as corticosteroids at 1 mg/kg/day for 3 to 4 weeks, tapered gradually over a period of 10 weeks to 1 mg/kg every alternate days and methotrexate at 7.5 mg weekly with gradual increase to 25 mg weekly for a period of 1 year when he experienced slightly improved muscle power.

**Figure 1:**
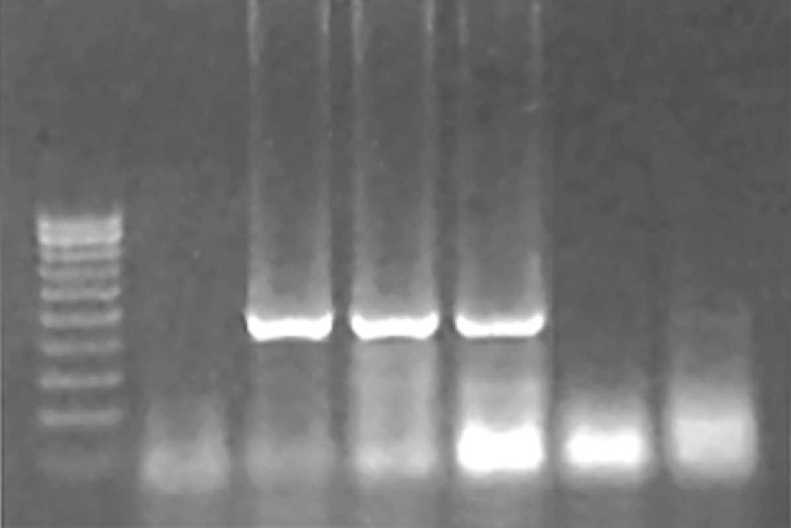
The PCR amplified product for *devR *gene (513 bps) of *M. tuberculosis*. (From left to right) Lane M; 100 bp ladder Banglore Genei (India), Lane 1; Negative control, Lane 2; *M. tuberculosis *H37Rv, Lane 3; Patient's isolate of sensitive *M. tuberculosis *strain (on first admission), Lane 4; Patient's isolate of MDR *M. tuberculosis *strain (on readmission), Lane 5; *M. kansasii*, Lane 6; *M. scrofulaceum*.

He had single cutaneous abscess 6 months later on the right side of the neck with no sinus formation and cervical and axillary lymphadenopathy. He was HIV-negative with normal CXR and there was no AFB in three consecutive (spot-early morning-spot) sputum samples (induced in two occasions). However, the aspirated pus from cutaneous abscess and biopsy of the axillary lymph node showed plenty of *M. tuberculosis *as culture was confirmed by niacin and nitrate reduction tests, rapid bacteriophage assay (FAST *Plaque *Tuberculosis kit, BIOTEC Laboratories Ltd, UK) and RFLP analysis (figure 1) using species-specific probes for *devR *(Differentially expressed virulent gene) [5].

In RFLP analysis, the *devR *gene (Gene bank nucleotide sequence accession no. U22037) encodes a response regulator that is part of a two-component signal transduction system of *M. tuberculosis*. The authenticity of the amplified product was established by hybridization of immobilized PCR products to an internal oligonucleotide, *devR1*, mapping within the *devR *gene. The chromosomal DNA was prepared by chloroform-isoamyl alcohol DNA extraction and 4.5 μg of DNA from the isolate was restricted with *Pvu*II. Separation of *Pvu*II-restricted DNA by electrophoresis, Southern blot hybridization with a 513-bp PCR probes for *devR *(*devRf*, 5' GGTGAGGCGGGTTCGGTCGC 3'; *devRr*, 5' CGCGGCTTGCGTCCGACGTTC 3') and chemiluminescence detection were done according to the standard method recommended for the DNA fingerprinting of *M. tuberculosis *[6]. Histopathologically there were nonspecific necrosis with disintegrating polymorphs, very few granulomatous cells and no epithelioid cells in the biopsy specimen. USG of kidney, liver or spleen revealed no abscess. The strain was susceptible to the 4 first line drugs [isoniazide (H), rifampicin (R), ethambutol (E) and pyrazinamide (Z)] by BACTEC 460TB system. Treatment with 4-drug regimen (H = 300 mg/d, R = 450 mg/d, E = 800 mg/d and Z = 1.5 g/d) for 8 months (2EHRZ/6HR) [7] with first 2 months supervised showed clinical improvement.

However, the patient returned 6 months later with multiple cutaneous abscesses, mainly on his back, left thigh and left arm and miliary mottling in CXR. He denied any irregularity in antituberculosis therapy. All immunosuppressive drugs (corticosteroids and methotrexate) were discontinued as he was readmitted. Isolates from 2 of 3 consecutive sputum samples and aspirated pus samples from all 5 completely drained abscesses were reconfirmed as the identical *M. tuberculosis *strain by phage assay and RFLP analysis (figure 1). The isolate was resistant to 4 first-line drugs by BACTEC 460TB system and to 9 drugs, tested into Loewenstein Jensen media (Hi-media, Mumbai, India): H (1 μg/ml), H (10 μg/ml), R (20 μg/ml), R (50 μg/ml), E (2 μg/ml), E (10 μg/ml), Z (50 μg/ml, at pH 5.5), streptomycin (5 μg/ml), streptomycin (50 μg/ml), paraaminosalicylate (2.5 μg/ml), cycloserine (30 μg/ml), amikacin (700 μg/ml) and ciprofloxacin (12.5 μg/ml). Treatment was continued with H (5 mg/kg/d), R (10 mg/kg/d), Z (30 mg/kg/d), E (15 mg/kg/d), kanamycin (15 mg/kg IM 5 times weekly) and sparfloxacin (500 mg/d) for 18 months with first 2 months under supervision. A slow but complete clinical, microbiological and radiological cure after 1.5 year was followed up for another 16 months.

## Discussion

India is an endemic country for TB with an estimated 20,000 infectious cases and 1–3.3% of new cases of MDR TB every year [8]. Infections are common with more than one strain of *M. tuberculosis *during the same episode (multiple infection), in different lesions (multiple infection) or during successive episodes (reinfection) in HIV-positive as well as -negative individuals [9]. In this study identical strains of *M. tuberculosis *were isolated at 6 months interval, but contrary to the first, the strain in the second episode was resistant to all the first line and most of the second line antitubercular drugs. As far as could be ascertained, this is the first case of isolation of MDR strain of *M. tuberculosis *from a HIV-negative but immunosuppressed patient who had an infection with the same, but susceptible strain 6 months before.

As evident from the study, the chance of exogenous reinfection with the same but drug-resistant strain from some undetected source within the endemic community is the most likely explanation. This explains the acquisition of resistance against all those drugs, to which the patient was not exposed earlier. Reinfection though occurs usually after first two to five years in immunocompetent hosts, may progress to active disease at any time after treatment has been discontinued and even during treatment for active tuberculosis [10]. Moreover, an ongoing tuberculous infection and simultaneous immunosuppressive therapy might significantly divert the immune response, thereby increasing the overall susceptibility to 'superinfection' [10] with the same but MDR strain. The chances of 'simultaneous infection' [11] with both the strains (susceptible and MDR) could be another possibility. Drug-susceptibility testing could discriminate simultaneous infection with different susceptibility profiles if individual colonies from the isolate were tested whereas in our case the sensitivity pattern of the susceptible strain might have been obtained at first attempt. Even RFLP and phage typing were not enough to determine simultaneous infections or reinfection (superinfection).

Endogenous reactivation which is higher than the rate of exogenous reinfection in endemic countries [12,13] with multi- "drug resistance in previously treated case"[14] might be a remote possibility. The history of regular medication itself might be notoriously misleading in patients with multidrug resistance [14]. However, it is difficult still to explain the acquisition of multidrug resistance of the strain in endogenous reactivation in such a short period against those drugs, to which the patient was not exposed. Any switch in specimens or cross-contamination of cultures in laboratories were unlikely since no other sample to switch or contaminate was identified.

Niacin test (95% positive [15]) and nitrate reduction test (97% positive [15]) differentiated *M. tuberculosis *from other mycobacteria in *M. tuberculosis *complex whereas RFLP and phage typing excluded atypical mycobacteria, like *M. kansasii *and *M. scrofulaceum*. The overall acuracy of the drug susceptibility test ranges between 84–100%, since mycobacteria often clump [16]. We tried thorough vortexing with glass beads to obtain homogenous inoculum suspensions.

It was a rare case of miliary TB of non-reactive type [17] with MDR *M. tuberculosis *invasion of the skin without any muscle involvement and sinus formation as the biopsy of the axillary lymph node showed non-specific necrosis containing disintegrating polymorphonuclear leukocytes and enormous number of AFB. This type is more often seen with severe HIV infection than in patients with immunosuppressive therapy [18,19], where the liver and the spleen are most commonly involved followed by the lung, the bone marrow and the kidney [17], granulomas and epithelioid cells are lacking and CXR shows inconspicuous diffuse mottling. In our case, there was no other organ involvement. Moreover, the rapid skin and lung involvement despite adequate antituberculosis therapy suggests that the tubercle bacilli were somehow protected from the drugs in the cutaneous and subcutaneous tissue ('paradoxical expansion of disease during therapy' [20]) either due to polymyositis-associated subcutaneous calcification or due to obstruction in the small arteries with the large mass of bacilli leading to necrosis and abscess formation in the surrounding areas.

This is probably the first reported case of cure where all first and most of the second line drugs (a total of 9 antitubercular drugs) were found to be resistant. However, the 6-drug antituberculosis therapy might not be considered as the only reason for cure, since 4 first line drugs showed high-level resistance and chances of cross-resistance between closely-related aminoglycosides, amikacin and kanamycin and quinolones, sparfloxacin and ciprofloxacin could not be ignored [21,22]. Discontinuation of immunosuppressive therapy with a reconstitution of the immune system and complete surgical drainage of the abscesses might have proved to be an important adjunct to the treatment.

## Conclusions

Severe immunosuppression may lead to disseminated TB such as miliary TB or other rare types of extra-pulmonary TB such as cutaneous abscesses. Follow-up of patients is important, and if response to treatment is poor, adherence to treatment, drug resistance and other possible reasons such as continuation of immunosuppressive therapy should be considered. In this case intervention by drainage of abscesses, discontinuation of immunosuppressive treatment and possibly long-term treatment with additional second line antituberculosis drugs eventually lead to cure.

## Abbreviations

AFB – Acid fast bacilli

AIDS – Acquired immunodeficiency disease syndrome

CXR – Chest radiograph

DNA – Deoxyribonucleic acid

EMG – Electromyography

HIV – Human immunodeficiency virus

MDR – Multidrug resistant

*M. tuberculosis*- *Mycobacterium tuberculosis*

NCV – Nerve conduction velocity

PCR – Polymerase Chain Reaction

RFLP – Restriction-fragment length polymorphism

TB – Tuberculosis

UK – United Kingdom

USG – Ultrasonography

WHO – World Health Organisation

## Competing interests

The author(s) declare that they have no competing interests.

## Authors' contributions

CM conceived the study, carried out the case study and follow-up, in the clinical as well as microbiological aspects, formatted the study design, performed sensitivity of the organism, participated in the FAST *plaque *assay and molecular identification method and drafted the manuscript.

AG carried out FAST *plaque *assay and molecular identification method.

AA participated in the design of the study and acted as an overall supervisor.

All authors read and approved the final manuscript.

## Pre-publication history

The pre-publication history for this paper can be accessed here:
